# Juvenile Steller sea lion (*Eumetopias jubatus*) utilization distributions in the Gulf of Alaska

**DOI:** 10.1186/s40462-018-0124-6

**Published:** 2018-05-15

**Authors:** Amanda Bishop, Casey Brown, Michael Rehberg, Leigh Torres, Markus Horning

**Affiliations:** 1 Alaska SeaLife Center, 301 Railway Avenue, Seward, AK 99664 USA; 2 Alaska Department of Fish and Game, 333 Raspberry Road, Anchorage, AK 99518 USA; 3 Marine Mammal Institute, Department of Fisheries and Wildlife, Oregon State University, 2030 SE Marine Science Dr, Newport, OR 97365 USA

## Abstract

**Background:**

A utilization distribution quantifies the temporal and spatial probability of space use for individuals or populations. These patterns in movement arise from individuals’ internal state and from their response to the external environment, and thus can provide insights for assessing factors associated with the management of threatened populations. The Western Distinct Population Segment of the Steller sea lion (*Eumetopias jubatus*) has declined to approximately 20% of levels encountered 40 years ago. At the height of the decline, juvenile survival appeared to be depressed and currently there is evidence that juvenile mortality due to predation may be constraining recovery in some regions. Therefore, our objectives were to identify what spaces are biologically important to juvenile Steller sea lions in the Kenai Fjords and Prince William Sound regions of the Gulf of Alaska.

**Methods:**

We examined geospatial location data from juvenile sea lions tagged between 2000 and 2014 (*n* = 84) and derived individual and pooled-population utilization distributions (UDs) from their movements. Core areas were defined from the UDs using an individual-based approach; this quantitatively confirmed that all individuals in our sample exhibited concentrated use within their home range (95% UD). Finally, we explored if variation in UD characteristics were associated with sex, season, age, or region.

**Results:**

We found evidence that individual juvenile home ranges were region and sex-specific, with males having larger home ranges on average. Core space characteristics were also sex-specific, and exhibited seasonal patterns of reduced size, increased proximity to haulouts, and increased intensity of use in the summer, but only in the Kenai Fjords-Gulf of Alaska region.

**Conclusions:**

This study highlights the areas of biological importance during this vulnerable life history stage, and the demographic, seasonal, and spatial factors associated with variation in movement patterns for a marine mesopredator. This can be useful information for promoting species recovery, and for future efforts to understand ecological patterns such as predator-prey interactions.

**Electronic supplementary material:**

The online version of this article (10.1186/s40462-018-0124-6) contains supplementary material, which is available to authorized users.

## Background

The movements of juvenile marine megafauna are understudied relative to adults [1]; however, it is becoming increasingly evident that there is variation in behavioral and ecological strategies during this vulnerable life-stage [2–4], which can have significant effects on fitness and survival [5]. Characterizing juvenile space use can therefore provide the foundational knowledge needed to address key questions in marine movement ecology such as the role of learning, memory and innate behaviors during ontogeny, the effect of predation risk on movement strategies, and the impacts of climate change on animal movement [1].

One way to characterize space use is a utilization distribution. Utilization distributions describe the finite space in which animals rest, forage, shelter, and reproduce [6], while also quantifying the spatial and temporal variation in the probability of use within the home range [7–9]. When concentrated use is exhibited within a home range, it is described as the animals’ ‘core space’ [10]. The characteristics of a utilization distribution may be influenced by an individual’s state (e.g. sex, age, body mass) and the external environment (e.g. conspecifics, habitat, prey density) dynamically interacting to influence an individual animals’ movement path [8]. As such, both home range and core spaces can vary in size, spatial pattern, or structure across temporal scales, regions, age-classes, or sexes [8, 11–13]. For example, beluga whales (*Delphinapterus leucas*) in the Eastern Beaufort and Eastern Chukchi Seas exhibited seasonal variation in the size and distribution of their home ranges [14] and male Eurasian lynx (*Lynx lynx*) had larger home ranges than females in all seasons [15].

Utilization distributions have been used to examine patterns of habitat use for residential and migratory animals [11, 16, 17], to assess predator-prey and territorial dynamics by comparing the home range and core spaces of two conspecific species [18], and to assess the potential for disease spread [19]. They also have been used to spatially and temporally identify where animal movements and human activities overlap [11, 20]. For example, the foraging utilization distributions of female New Zealand sea lions (*Phocarctos hookeri*) showed extensive overlap with fishery operations [20], and the home range of Indo-Pacific bottlenose dolphins (*Tursiops aduncus*) in sheltered waters had a high degree of overlap with human activity [11]. Habitat degradation, predation, disease, bycatch, and disturbance can all influence survival and/or reproductive output [21, 22]; therefore, where management of a listed species or a vulnerable age-class is implemented by way of spatial or regional resource use regulations, characterizing the utilization distributions of individuals and populations can provide important information for management decisions [17, 23].

Steller sea lions (*Eumetopias jubatus*) are the largest of the otariid pinnipeds. Based on demographic and genetic differences, Steller sea lion populations have been designated into two distinct population segments, of which the western Distinct Population Segment (wDPS west of 144° W) in the North Pacific is listed as endangered under the Endangered Species Act (USFR: 62:30772–30,773). Starting in the late 1970s, the wDPS has declined to approximately 20% of levels encountered 40 years ago [24]. While currently some regions of the wDPS, such as the Gulf of Alaska, seem to be stable or slightly increasing [25], it has been suggested that the continued decline in the western Aleutian Islands and the lack of recovery for the wDPS as a whole might be driven by killer whale predation [26] or inadequate food resources [27]. At the height of the decline, juvenile survival appeared to be depressed [28–30] and recent telemetry studies [31, 32] have revealed that predation accounted for 91.7% of mortalities (95% CI: 78–100%) in juvenile SSL in the Prince William Sound and eastern Gulf of Alaska region.

The behaviors of juvenile Steller sea lions in the Gulf of Alaska (wDPS) have been investigated previously, but efforts primarily focused on characterizing diving behaviors and ontogenetic changes in diving in the first year [33, 34], haul-out behaviors [35, 36], and movements between haulout complexes [4]. These studies have provided key insights into the physiological constraints and connectivity dynamics for Steller sea lions but when considering the species’ recovery, it may also be important to consider spatial overlap and encounter probabilities between predators and prey for this age-class [1, 18, 37]. Characterizing the utilization distributions of juveniles would be an important first step; yet, only one published study has quantified the home ranges for Steller sea lions, and this work focused on comparing adult and young of the year (< 1 year old) animals from Kodiak Island (57.5°N, 153.5° W) [38].

Therefore, our objectives were to: (1) develop population, and individual-based utilization distributions from telemetry-derived locations from juvenile Steller sea lions in two regions within the wDPS, Kenai Fjords and Prince William Sound, (2) from these, identify and characterize home range and core spaces, and (3) explore if variation in space use patterns is associated with sex, season, age, or region. By taking a holistic and individual-based approach to characterizing space use, we will gain a better understanding of a vulnerable age class in this endangered species.

## Methods

### Animal captures

**Fig. 1:**
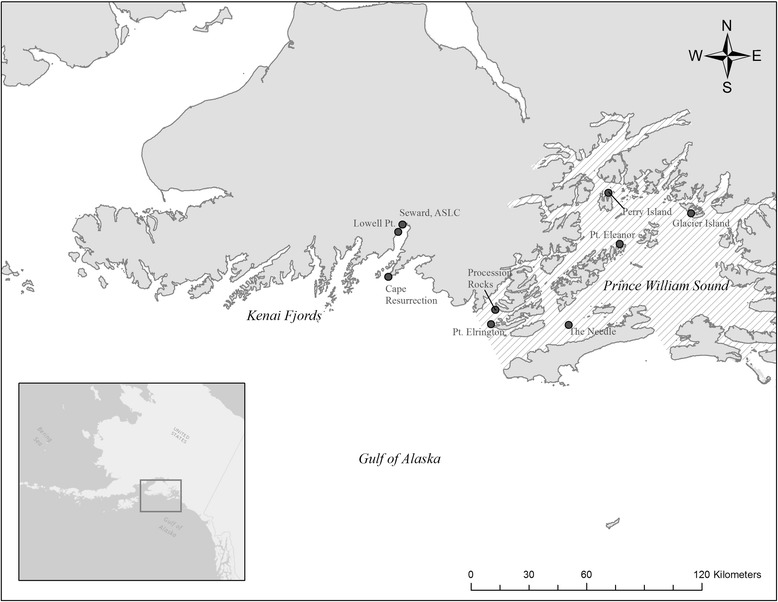
Sites where juvenile Steller sea lions (*Eumetopias jubatus*) were captured and/or released in Kenai Fjords and Prince William Sound*.* Hashed background represents boundary of Prince William Sound Groundfish Statistical Area interior district, accessed from Alaska Department of Fish and Game [48]*. Map datum is NAD83, projected to North American Albers Equal Area Conic*

The capture, handling, and instrumentation of weaned juvenile Steller sea lions (SSLs) included in the present study (*n* = 88; 49 male, 39 female) is described in Call et al. [36], Raum-Suryan et al. [4], Mellish et al. [39, 40], and Thomton et al. [41]. Briefly, animals were captured between 2000 and 2014 by the Alaska Department of Fish and Game (n = 8) and the Alaska SeaLife Center (*n* = 80) in Prince William Sound (60° N 148° W) and Resurrection Bay, Alaska (60^0^ N 149.3^0^ W, Fig. 1). After capture, animals were transferred to a research boat, immobilized with gas anesthesia [42], and weighed to the nearest 0.1 kg. Age estimation was based on a combination of body mass, tooth eruption patterns, and time of year [43], with juveniles defined as animals > 12 mo. SSLs were either released near the capture site (“Free Ranging, FR”, *n* = 21), or transported to the Alaska SeaLife Center for temporary captivity lasting up to 3 months (“Transient, TJ”, *n* = 67). Transient animals were part of multiple research projects and were subject to various health and veterinary assessments [39]. A subset of these animals (*n* = 45) underwent surgery for implantation of life history tags, telemetry devices that provide information on vital rates and mortality [44–46]. Implanted animals were monitored in captivity for periods of 1–6 weeks after surgery and before release at Lowell Point in Resurrection Bay [40, 41, 45]. Temporary captivity was found to have no significant effect on animal diving behavior post-release [40, 41], physiological parameters were the same between free ranging and transient animals [39], and there was no evidence of reduced survival due to LHX implantation surgery or temporary captivity [47]. Therefore, the movement data from all Transient and Free Ranging juveniles were included in the same manner for this study, but a post-hoc test was conducted to assess the effects of handling treatment (TJ *n* = 16, vs FR *n* = 17; see below).

### Location data

In all cases, prior to release SSLs were instrumented with an external satellite data recorder (SDR). The telemetry device (either a SDR-T16, SPOT 5, or SPLASH, Wildlife Computers, Inc.) was glued to the dorsal pelage along the midline of the back, in alignment with the fore-flippers [40, 41]. SDRs used saltwater immersion sensors to determine when the antenna was not submerged. Once submerged for 4 or more consecutive transmission intervals after an extended dry period, tags transmitted as soon as the surface was breached, at a repetition rate not exceeding approximately 45 s. After 10 consecutive dry transmissions, tags switched to a slower repetition rate of approximately 90 s. After a preset number of hours, the tags ceased all transmissions until once again submerged. For different groups of animals within the study, 6 and 12 h presets were used. Tags transmitted until shed in the annual molt, or until the tag malfunctioned or ran out of battery power. Argos system service provider CLS America received SDR transmissions and provided location estimates derived from an analysis of the Doppler shift frequency data from multiple sequential transmissions received during a single satellite pass [49]. Locations were assigned an accuracy estimate ranging from < 150 m (LC 3) to > 1.5 km (LC B) by CLS America. As we were interested in developing a holistic view of individual variation in juvenile space use and in quantifying the total utilization distribution of juvenile Seller sea lions, we included all locations on land and at sea in our analysis.

Raw data was decoded by the Wildlife Computers Data Portal. Locations were processed using several steps to remove locations without an assigned accuracy (LC Z) and to interpolate points in time and space. First, to remove extreme outliers and meet normality assumptions for subsequent state space models, we omitted all locations with LC Z [50] and applied a swim speed filter of ≤8 m^-s^ (estimates of mean velocity for this species range from 2.7–3.4 m^-s^, [38, 51, 52]. We further truncated any tracks to remove any > 4 day gaps, as these could cause off-shoots inland, across land, or loops when run through the subsequent state space model (*n* = 15 tracks, *n* = 248 points removed, or 0.4% of total data). This filter provided more confidence that we captured the true end of a track, and not data from the tag transmitting after detachment from an animal. Individuals’ filtered paths were then interpolated to generate pseudolocations at equal intervals (2 h, 12 locations per day) using a continuous time correlated random walk in package *crawl* [50]. This process was repeated 500 times per track, and the generated pseudolocations were averaged across all simulations for a single track per individual.

To account for cases where pseudolocations were erroneously assigned to land due to the regional geography, we ran the final averaged path for each individual through {*fixpath}* in *crawl,* a function which moves points on land to the closest sea location along the path trajectory. This process also moves pseudolocations where an animal may have accurately been hauled out into the water. In these cases, the new pseudolocations would be < 1 km from the haulout location when included in UD calculations. This adjustment would therefore still provide a representation of the space use near a haulout, but would not differentiate whether the animal was on land or in the water around the haulout.

### Utilization distributions

Utilization distributions (UDs) calculate an index of residence probability per unit area. Population-level UDs provide a spatial extent that may be useful for management decisions that are spatially or temporally explicit [53]. Therefore, for all 84 individuals’ pseudolocations pooled together (*n* = 72,817), a UD was calculated using a tracking-weighted fixed-kernel density analysis [16, 20]. Kernel density estimates (kde) were generated across a 1 km × 1 km grid using a fixed likelihood cross-validation bandwidth (Geospatial Modeling Environment, GME v0.7.2.0). To account for the potential spatial bias associated with a large number of individuals in the pooled sample being released from the same location (Additional file 1: Table S1), we applied a weighting approach that has been utilized to adjust probability grids for other pooled-samples of marine mesopredators [16, 20]. This involved weighting the kde grid by the total number of individual sea lions that were observed in each cell to reflect equal sampling effort, and generate an effort-corrected UD [16, 20]. For individual comparisons, kernel density grids (1 km × 1 km) were generated separately for individuals’ locations within six biologically relevant seasonal periods: Jan-Feb (*n* = 25), Mar-April (*n* = 19), May–June (Pupping, *n* = 34), July-Aug (Breeding/Molting *n* = 29), Sept-Oct (*n* = 22), and Nov-December (*n* = 35) using a fixed likelihood cross-validation bandwidth (GME v0.7.2.0). Prior analysis of this dataset found no relationship between UD area and the duration of tag deployment or the number of fixes [54], and UDs have been calculated for other pinnipeds from as few as 2–3 days of tracking [20]. Therefore, in order to meet the minimum sample size requirements of kernel density analysis and ensure a reasonable representation of space use without bias from interpolation [55], only bi-monthly samples with > 50 pseudolocations, and where the pseudolocation to raw location ratio was < 3 were included in analysis of individual UDs (Additional file 1: Table S1). Note that some individuals contributed to more than one bi-monthly periods. This resulted in the removal of another 4 individuals, for a final dataset of 164 bi-monthly kernel density grids originating from 84 individuals.

For both effort-corrected pooled and individual kernel density grids, the 95% UD isopleth was selected to define the boundary of the home ranges [9, 11]. Home range polygons were clipped to exclude land, and the area (km^2^) of the home ranges were calculated using standard tools in ArcGIS 10 (ESRI). While many studies define highly utilized space, or the “core” within a home range, as the 50% or 25% isopleth [16, 56, 57], we selected to use an individual-based quantitative approach to calculate core [9]. We fit an exponential regression to a plot of UD area (km^2^) against isopleth volume and determined the point at which the slope of the line fitted was equal to 1 [9]. This point represents a limit where the home range area begins to increase at a greater rate than the probability of use, and the corresponding isolpeth volume defines the boundary of the core space. We used this approach to define core space for the effort-corrected pooled population, and for each individual’s bi-monthly UD. This method further enabled us to verify the existence of core area for the population, and seasonally for individuals, by calculating the relative intensity of use index (*I*) as the ratio of the isopleth volume of the core boundary, to the percent of the home range area the core space area occupied [9, 10]. Values of *I* < 1 indicate no difference between core space and home range utilization, or no existence of a core. The total number of core polygons, and the total area (km^2^) of all core polygons were calculated for the effort-corrected pooled population and for each individual bi-month UD, using ArcGIS 10 (ESRI). Using the centroid of each core polygon, and SSL haulout locations in Alaska [58], we also calculated the minimum Euclidean distance from a core space to a haulout for each individual in a bi-month period as a measure of proximity to resting habitat. Values are reported as means with standard error in parentheses.

### Analysis

Using the UDs generated for individuals, generalized additive mixed-effects models (GAMMs) were used to examine factors associated with variation in juveniles’ space use metrics (*n* = 164). Our space use response variables included (1) home range area (km^2^), (2) core area (km^2^), (3) number of haulouts within home range, (4) intensity of use (*I*), and (5) minimum distance to a haulout (km). To meet normality, home range size, core area size, and intensity of use were log-transformed, minimum distance to a haulout was square-root transformed, and the model for number of haulouts within home range had a Poisson distribution (link = log). Our predictor variables included sex, season (6 bi-month periods), and region. Region was defined by whether the home range was entirely in Prince William Sound (PWS, Fig. 1), entirely outside of PWS: Kenai Fjords-Gulf of Alaska (KFGOA), or spanned both. We also included interactions between season and sex, season and region, and sex and region to further consider variation in space use patterns. We could not include a three-way interaction between season, sex and region due to sample sizes. We selected to use GAMMs, in R package *gamm4* [59] with individual ID as a random effect. As months exist on a temporal circle with no endpoints, we needed to account for the cyclical nature of our temporal factor, bi-month period. The GAMM enabled us to include season as a smoothed factor with a cyclic cubic regression spline, which constrains the pattern at the end of the cycle (November–December) to carry through to the beginning of the cycle (January–February). Separate models were run for each of our four response variables.

We also wanted to investigate any changes in space use from juveniles in their first year after weaning (12–23 mo) to those in their second year (24-35mo). As annual synchronous breeders, age is interlinked with season for many pinnipeds, including SSLs. Therefore, we investigated the effect of age by only looking at the bi-month periods in which we had both 1 and 2-year-old individuals present (MJ, JA, SO, *n* = 85). These models, hereafter referred to as ‘truncated’, had the same response variables and included all previous predictor variables, with the addition of age (Y1 or Y2), and interactions with age. These were fit as generalized linear mixed-effects models, GLMM in R package *lme4* [60] with ID as a random effect, as season was no longer cyclical.

For each response variable, model selection followed the criteria of Richards [61] in which AIC is calculated for all simpler versions of the most complex model (global model). The final model set then included models with ΔAIC < 6 that were not nested versions of simpler models. These criteria prevent the selection of overly complex models [61]. For each model, relative importance scores were calculated for each predictor variable as the sum of model weights of all models in the final model set which contained that variable [62]. Based on the final model results, we used an ANCOVA test to compare home range area across handling treatment (TJ/FR), sex, and an interaction between treatment and sex for individuals in PWS (the region where most FRs were tagged).

## Results

Juvenile SSLs were tracked for an average of 77 days ±5.74, resulting in 72,816 pseudolocations generated across the 84 individuals (866.6 ± 68.45 per ID) [Additional file 1: Table S1].

### Utilization distribution characteristics: Pooled juveniles

**Fig. 2:**
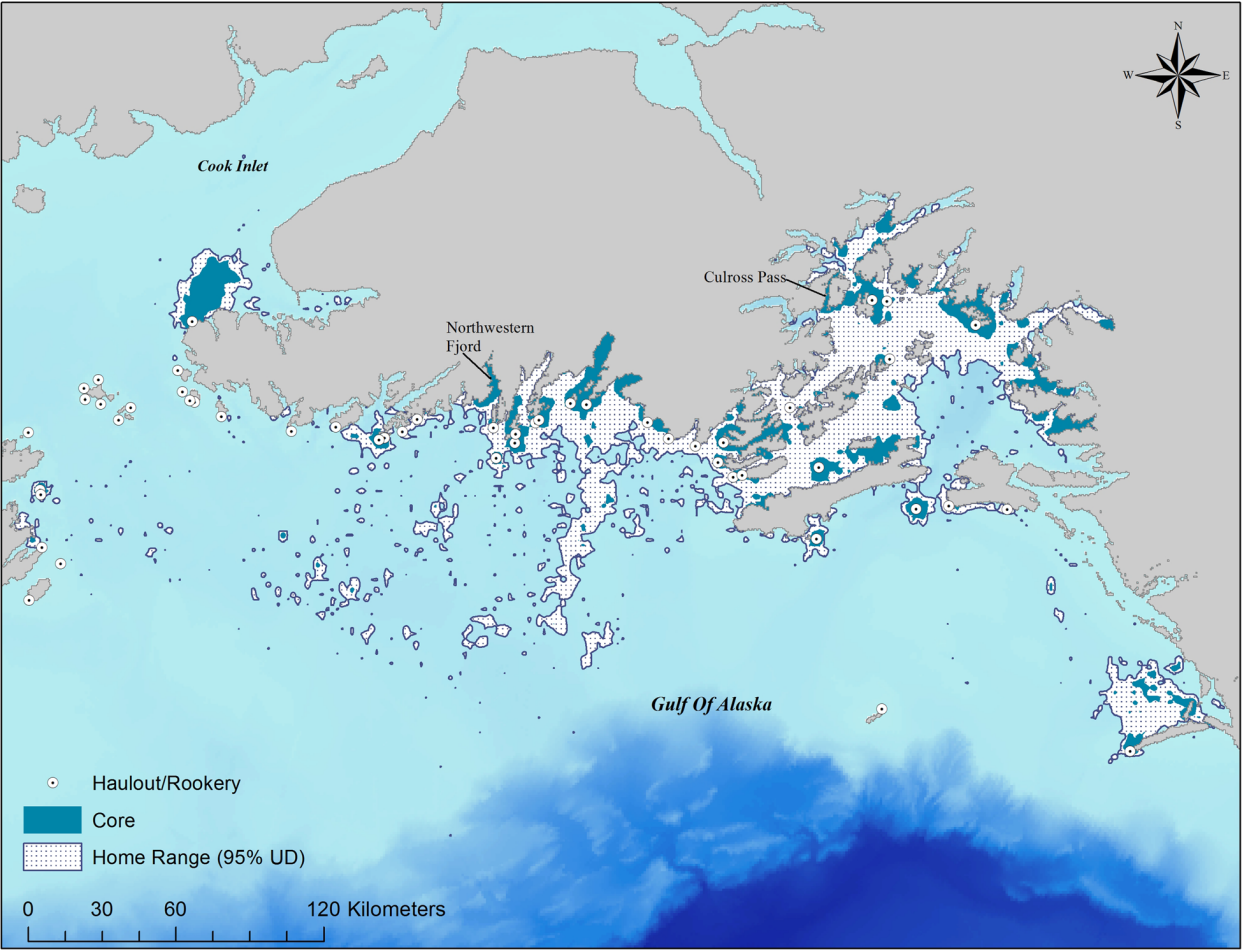
Effort-corrected utilization distributions (95% Home Range, and nested core space) for pooled tracking data (*n* = 84 individuals) with the known Steller sea lion haul-outs and rookeries in those regions [57]. *Map datum is NAD83, projected to North American Albers Equal Area Conic*

In general, the pooled-juvenile home range extended from Kayak Island in the east (59.9^0^ N, − 144.4^0^ W) to Kodiak Island in the west (58.2^0^N, − 154.3^0^W), and was generally coastal, with some evidence of excursions offshore onto the shelf, or to adjacent regions. The effort-corrected utilization distributions of pooled tracking data between 2000 and 2014 resulted in a large 95% UD home range totaling 12,005.2 km^2^ (Fig. 2). Within the home range, core space for the population was identified as the 64.4 isopleth volume (residual standard error = 0.012). This resulted in a core space area of 2799.4 km^2^, which accounted for 23.31% of the area of the home range. Intensity of use in in the core space, relative to the home range, was 2.76 times greater. The core space was comprised of 177 discrete polygons (15.81 km^2^ ± 3.3), many of which were associated with primary Steller sea lion haulouts or rookeries (Fig. 2).

### Utilization distribution characteristics: Individual juveniles’ seasonal patterns

**Fig. 3:**
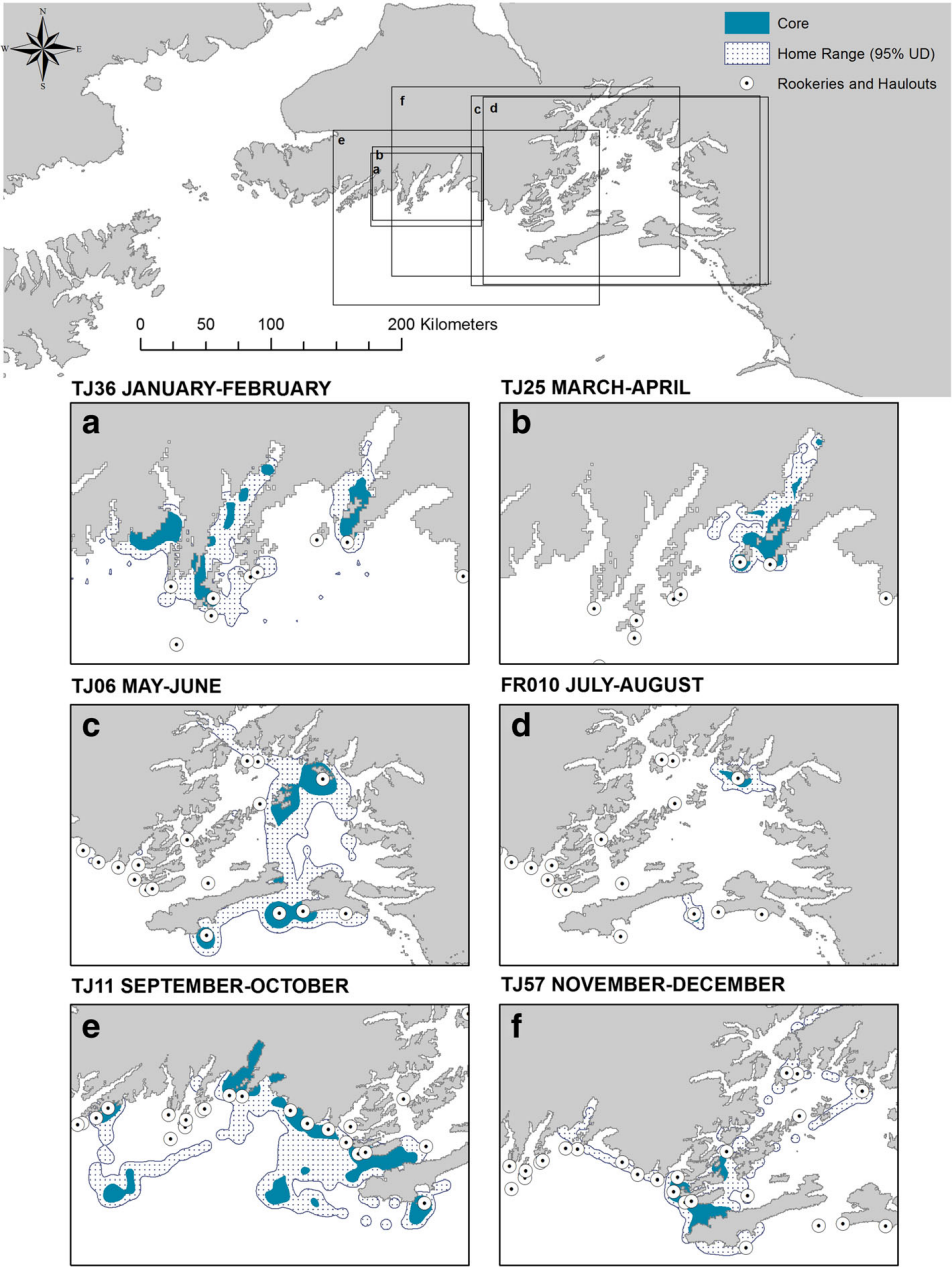
Examples of 6 individual juvenile Steller sea lions’ core UDs nested within 95% UD (Home Range); examples span each of the 6 bi-monthly periods (**a**) JF = Jan-Feb, (**b**) MA = Mar-Apr, (**c**) MJ = May–June, (**d**) JA = July = Aug, (**e**) SO = Sept-Oct, (**f**) ND = Nov-Dec. *Rookery and haulout location data* [57]*. Map datum is NAD83, projected to North American Albers Equal Area Conic*

Individual juvenile SSL home ranges in bi-monthly periods (95% UD) varied in their distribution, size, and shape (Table 1, Fig. 3). Within individuals’ home ranges, there were clear core-spaces, sometimes comprised of multiple discrete polygons (Table 1, Fig. 3). Isopleth volumes that defined core space were not significantly correlated with home range size (*p* = 0.813). Core space was on average designated at the 61.42% UD isopleth, but ranged from 31.62% to 71.83% (Table 1). Proportionally, core space accounted for on average 21.35% (± 0.96) of the home range size of individuals (Table 1), and the relative intensity of use in core space varied from 1.31 to 29.34 times more use than in the rest of the home range (Table 1). No individuals had intensity values < 1, suggesting all juveniles in this study had core space that could be differentiated from their home range.

**Table 1:** Summary statistics for individual juvenile Steller sea lion bi-monthly Utilization Distributions (*n* = 164 (across 84 IDs)), in the Gulf of Alaska, 2000–2014

	Mean	se	Min	Max
Home range area (km^2^)	851.72	157.76	7.83	15,886.18
Core area (km^2^)	166.54	34.03	0.94	3587.98
Home range perimeter (km)	454.77	40.29	19.87	2808.49
Proportion Core (%)	21.35	0.96	1.60	52.32
Isopleth Volume Core (%)	61.42	0.55	31.62	71.83
Residual Standard Error^α^	0.02	0.001	0.007	0.07
Percent of Positions in the Core	67.05	0.71	36.63	88.46
Number of Core Polygons	3.80	0.26	1	23
Distance from Centroid(s) to nearest Haulout/Rookery (km)^1^	22.61	1.76	0.00	125.40
Relative Intensity of Use (*I*)	4.47	0.32	1.31	29.34

*α: From regression of exponential curve fit*

*1: Haulout or rookery that has closest average distance to all centroid*s

### Home range characteristics

**Fig. 4:**
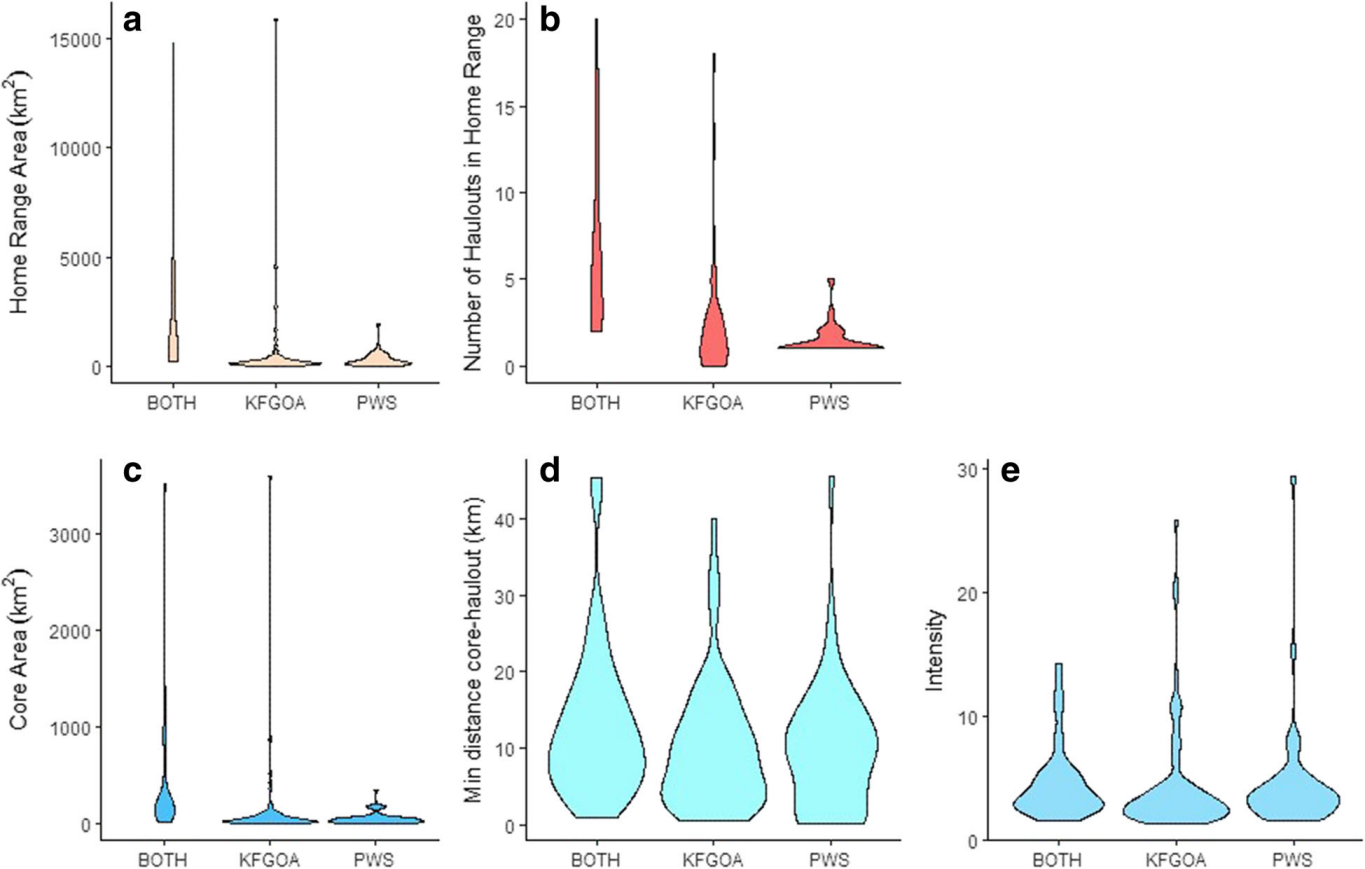
Frequency distributions of home range characteristics (home range area (**a**), and number of haul outs in home range (**b**)), and core space characteristics (core area (**c**), the minimum distance from core centroid to a haul out (**d**) and intensity of use of core relative to home range (**e**)) relative to region (if home range was exclusively in Prince William Sound: PWS, entirely within Kenai Fjords Gulf of Alaska; KFGOA, or if home range spanned BOTH) for juvenile Steller sea lions

The best model based on AIC for home range area included region and sex, with region having the higher relative importance (Table 2a, Table 3). Individuals that used both PWS and KFGOA had larger home ranges on average relative to animals that remained exclusively in PWS or exclusively in KFGOA; however some individuals in the KFGOA region also utilized home ranges > 2500 km^2^ (Fig. 4a). Male home-ranges tended to be larger (*n* = 104; 1080.1 km^2^ ± 239.3) relative to females (*n* = 60; 455.66 km^2^ ± 102.6), but this difference was not significant. When including age as a predictor variable, the best truncated model included season, region and sex (Table 2b). The importance of region was similar, but sex was relatively more important in the truncated model (Table 3). There was some evidence for a seasonal effect on home range in these bi-months (Table 2b). Home ranges in July–August (199.99 km^2^ ± 45.6) were smaller than home ranges in May–June (715.47 km^2^ ± 181.5) or September–August (1027.31 km^2^ ± 319.0). There was no age-effect in the best model (Table 2b). From our posthoc test, handling treatment (TJ vs. FR) had no significant effect on home range area for individuals in PWS (*F = 2.081*, df = 45, *p* = 0.1161).

**Table 2:** Summary of generalized additive models to predict home range characteristics

Response Variable	df	AIC	ΔAIC	weight
*(a) Home Range Area*				
Region + Sex	6	500.16	0.00	0.50
Region	5	500.33	0.17	0.49
*(b) Home Range Area (w/age)*				
Season + Region + Sex	8	252.959	0.00	0.61
Season + Region	7	255.269	2.31	0.24
Region + Sex	6	257.272	4.313	0.11
*(c) Number of Haulouts in home range*				
Region + s(Season:Sex) + Sex	7	434.99	0.00	0.42
Region + s(Season) + Sex	6	436.38	1.39	0.23
Region + s(Season:Sex)	6	437.02	2.03	0.17
Region + Sex	5	438.52	3.53	0.09
Region + s(Season)	5	438.82	3.83	0.07
*(d) Number of Haulouts in home range (w/age)*				
Season + Region + Sex	7	195.05	0	0.35
Season + Region + Age + Season:Age	9	195.10	0.05	0.22
Region + Sex	5	196.66	1.61	0.22
Season + Region	6	197.32	2.27	0.14
Region	4	199.16	4.11	0.07

Models presented are those included in our final model set based on criteria <Δ6 AIC and not a nested version of a simpler model [53]. Variables preceded by an *s* indicate the factor was included as a smoothed factor with a cyclic cubic regression spline

**Table 3:** A summary of models for each home range and core space characteristic with relative importance scores for 4 predictor variables (and 5 interactions)

Models GAMM	Region	Sex	Season	Region: Sex	Sex: Season	Region: Season	Age	Age: Season	Age: Sex	Age: Region
Home range area	1.00	0.50	0.00	0.00	0.00	0.00	*	*	*	*
#Haulouts in home range	0.98	0.91	0.31	0.00	0.59	0.00	*	*	*	*
Core area	1.00	0.00	0.00	0.00	0.00	0.50	*	*	*	*
Min. distance core to haulout	0.93	0.70	0.07	0.70	0.00	0.85	*	*	*	*
Relative Intensity of Use, *I*	0.00	0.57	0.00	0.00	0.00	0.99	*	*	*	*
**Truncated Models w/ Age GLMM**										
Home range area	0.95	0.71	0.85	0.00	0.00	0.00	0.00	0.00	0.00	0.00
#Haulouts in home range	1.00	0.57	0.71	0.00	0.00	0.00	0.22	0.22	0.00	0.00
Core area	1.00	0.00	0.00	0.00	0.00	0.00	0.00	0.00	0.00	0.00
Min. distance core to haulout	0.88	0.00	0.88	0.00	0.00	0.88	0.00	0.00	0.00	0.00
Relative Intensity of Use, *I*	0.00	0.00	0.92	0.00	0.00	0.00	0.00	0.00	0.00	0.00

* indicated the variable was not included in the model. Models without age:season and interactions between sex:season and region:season are cubic-splines. Models with age utilized a truncated dataset, which only included data from May–October

Region, sex, and an interaction between season and sex were retained in the best model for predicting the number of haulouts in a home range, and region had the highest relative importance followed by sex (Tables 2c & 3). Home ranges for juveniles that spanned both regions encompassed the most haulouts (Fig. 4b). There was no difference on average between KFGOA and PWS individuals, but KFGOA individuals were more variable (Fig. 4b). Males had slightly more haulouts in their home range (3.7 ± 0.4) than females (2.1 ± 0.2), and seasonal differences were apparent for males only (Table 2c), in that males had the least haulouts per home range in March and April (*n* = 7; 2.38 ± 0.6). When including age in the truncated model, the regional and sex effects were retained (Table 2d); however, the relative importance of sex was reduced (Table 3). The best model also included a non-sex-specific seasonal effect (Table 2d), in which there were fewer haulouts per home range in July–August (1.83 ± 0.2) relative to September–October (5.09 ± 1.2, (Table 2d). Age was not retained in the best model (Table 2d); however, it did have some relative importance when considering the whole model set (Table 3).

### Core characteristics

**Fig. 5:**
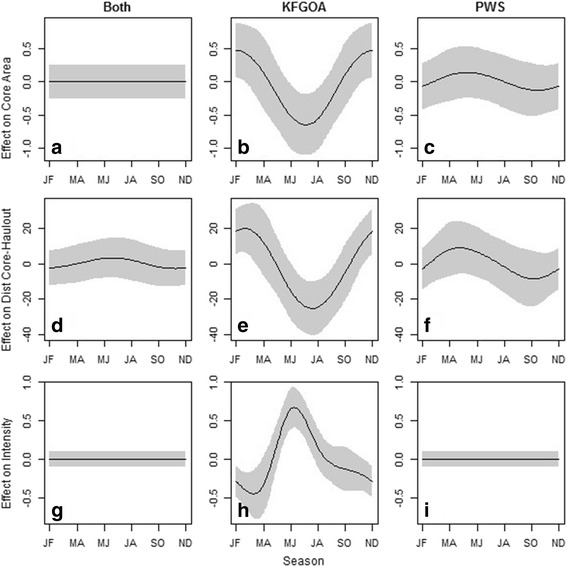
Functional response curves from generalized additive mixed effects model (GAMM) cubic splines showing the relative effect of seasonality (JF = January–February, MA = March–April, MJ = May–June, JA = July–August, SO = September–October, ND = November–December) in different regions (Both, KFGOA = Kenai Fjords/Gulf of Alaska, PWS = Prince William Sound) on animal core space characteristics (Core space area (**a-c**), Distance from Core-Haulout (**d-f**), Intensity (**g-i**)). Note y-axes differ by response variable

For the model examining core space area, region and an interaction between region and season were retained in the best model (Table 4a), with region having the highest relative importance (Table 3). Core spaces were largest for individuals that used both PWS and KFGOA; however, there were also some individuals in KFGOA region with large core space (Fig. 4c). While juveniles in KFGOA and PWS had similar sized core areas on average, juveniles in KFGOA exhibited a seasonal pattern, with core areas decreasing from May–August, then increasing into the winter (Fig. 5a). This seasonal interaction was not apparent for animals in PWS, or animals whose movements spanned both areas (Fig. 5b-c). There were no sex or general seasonal patterns in core area size (Table 4a). There was no effect of age on core space size in the truncated model, and only region was retained (Table 4b).

**Table 4:** Summary of generalized additive models to predict core space characteristics

Response Variable	df	AIC	ΔAIC	weight
*(a) Core Area*				
Region + s(Season:Region)	8	537.3	0.00	0.50
Region	5	537.9	0.60	0.49
*(b) Core area (w/age)*				
Region	5	278.35	0.00	1
*(c) Minimum Distance Core-Haulout*				
s(Season:Region) + Region + Sex + Region:Sex	11	1671.06	0.00	0.56
s(Season:Region) + Region	8	1673.63	2.57	0.23
s(Season) + Region + Sex + Region:Sex	9	1675.86	4.80	0.07
Region + Sex + Region:Sex	8	1676.43	5.37	0.07
S(Season:Region)	6	1676.51	5.45	0.06
*(d) Minimum Distance Core-Haulout (w/age)*				
Season + Region + Season:Region	11	840.54	0.00	0.88
*(e) Intensity*				
Sex + s(Season:Region)	8	293.80	0.00	0.58
s(Season:Region)	6	294.60	0.80	0.42
*(f) Intensity (w/age)*				
Season	5	168.04	0.00	0.92

Models presented are those included in our final model set based on criteria <Δ6 AIC and not a nested version of a simpler model [53]). Variables preceded by an *s* indicate the factor was included as a smoothed factor with a cyclic cubic regression spline

Region, sex, an interaction between region and sex, and an interaction between season and region were retained in the best model for the minimum distance of core space from haulouts (Table 3, Table 4c). In KFGOA, core spaces were closer to haulouts than the cores of individuals in PWS or for individuals that spanned both regions (Fig. 4d). Seasonal patterns were only apparent in KFGOA, with core spaces being closer to haulouts in May–August relative to other periods (Fig. 5d-f). Males’ cores were slightly farther from haulouts (11.02 km ± 0.9) than females (9.95 km ± 1.03), but in PWS only (Table 4), males’ core spaces were closer to haulouts (*n* = 17; 6.86 km ± 1.6) than females (*n* = 27; 11.92 km ± 1.7). There was no effect of age on core space distance from haulouts in the truncated model, and sex was no longer retained when only considering bi-months from May–October (Table 4d).

The best model for intensity of core space use retained sex and an interaction between region and season (Table 4e), with the interaction between season and region having the highest relative importance (Table 3). There was no difference in the average intensity for individuals in PWS, KFGOA or those that used both regions (Fig. 4e), but seasonal differences in intensity of use were apparent for individuals in KFGOA, with intensity increasing in May–June then declining through the fall and winter into March–April (Fig. 5g-i). There was a slight indication that males had higher intensity of use (4.61 ± 0.4) compared to females (4.23 ± 0.5). Age was not retained in the truncated model (Tables 4f, Table 3).

## Discussion

This study contributes to a better understanding of utilization distribution patterns of an understudied age-class, and the movement ecology of an endangered marine mesopredator. We identified the areas and periods of key biological importance for juvenile Steller sea lions in the Kenai Fjords, Gulf of Alaska, and Prince William Sound regions, and confirmed the presence of core space for this species and age-class. When exploring factors associated with the observed variation in individual utilization distribution characteristics, our results suggest that sex-specific patterns were present at both the scale of home range and core space, but that core space characteristics varied seasonally in some regions.

### Juvenile home ranges

When considering factors associated with variation in home range size and structure across individuals, our results suggested sex and region-specific differences, but lacked evidence of seasonal patterns associated with juvenile space use. Across regions, individuals that remained exclusively in Prince William Sound or Kenai Fjords/Gulf of Alaska had on average similar sized home ranges, which tended to encompass an average of 3–4 haulouts. Individuals that used both regions had the largest home ranges, and their home ranges encompassed a greater number of haulouts; however, some individuals who remained exclusively in the Kenai Fjords/Gulf of Alaska region also had equally large home ranges (Fig. 4a-b). Other central place foragers have also exhibited similar individual variation in the size and extent of home ranges, and these patterns are likely driven by individual strategies and/or experience [63]. For example, approximately half of juvenile northern fur seals (*Callorhinus ursinus*) visit other haulout sites and islands between foraging trips [64]. Adult wandering albatross (*Diomedea exulans*) also exhibit variable UDs and movements, with “sedentary” birds foraging in the waters near their breeding colony throughout the year and ‘migratory’ birds travelling long distances from their colony [63].

In addition to regional differences in home range, our results suggested sex-related differences in home range characteristics, with juvenile male Steller sea lions tending to have larger home ranges that encompassed more haulouts. This complements prior telemetry and mark-recapture studies on juvenile Steller sea lions that have shown evidence of post-weaning dispersal for males [4, 65]. For many species, males tend to utilize larger home ranges, travel farther, and dive deeper than females [15, 63, 65–67]. This pattern may be driven by males having greater energy requirements and experiencing greater competition for mates later in life, particularly in sexually size dimorphic or polygynous species [66, 67]. Females alternatively tend to exhibit smaller space use, potentially due to reproductive constraints [68, 69]. Age however was not found to be a significant cofactor for home range in our study for either sex. The age at first ovulation for Steller sea lions is on average 4.6 years for females [70], and sexual maturity is reached at 6 years for males, (though males are not typically competitive for territories until 9–13 years old), [71, 72]. Therefore, it is unlikely reproductive mechanisms would result in age-related differences in space use between 1 and 2 and 2–3 year old age classes. However, Steller sea lions exhibit development of diving ability during the first year of life through their juvenile stage [73], which could suggest that horizontal movements would also increase with age. We did not find evidence of this pattern, but monitoring the development of individual strategies regarding home range and core space utilization could provide novel insights into the process and fitness consequences of phenotype selection [74].

Due to the differential designations of the wDPS and eDPS, previous work explored how behaviors differed across populations of SSL experiencing different trajectories in an effort to inform management decisions and recovery plans [4, 75]. However, the only other published study that has quantified individual home ranges for Steller sea lions investigated the space use of 15 adult females and pups off the coast of Kodiak Island, in the central Gulf of Alaska, west of our study region (Fig. 1) [38]. The home range size for adult females in that study were on average 45,579 km^2^ in the winter, and 319 km^2^ in the summer, while pup home ranges in the winter were on average 9196 km^2^ [38]. For most pinnipeds, we might expect juveniles to exhibit larger home ranges than pups due to ontogenetic development of behavioral differences in swimming or diving [76–78]. However, our results suggested that the home ranges for juveniles in the eastern Gulf of Alaska were an order of magnitude smaller, averaging 851 km^2^ across all seasons. While in general, pups and adult females had larger home ranges in the Merrick and Loughlin [38] study, they noted that for the five pups they tracked, only one individual exhibited the 36,320 km^2^ home range, and that most used areas ranging from 1000 to 4000 km^2^. Even with the removal of the potential individual outlier, the juveniles in the present study were still using smaller home ranges on average. A potential limitation to direct comparison between our results and other studies is the difference in methods utilized to calculate individual home ranges. The previous work on SSL defined home ranges with minimum convex polygons (MCPs), which can over-estimate space use in the presence of outliers [79]. Similarly, direct comparison might be difficult to other studies of pinniped UDs that incorporate only at-sea locations [20, 53], or those that utilize Fastloc GPS to generate locations [80]. Accounting for these differences, future comparisons of our findings to complementary assessments of home range and core space in other regions of the Steller sea lion range, or to other populations of pinnipeds, could provide insights into how juveniles are differentially responding to their environment, and the potential constraints of recovery in declining populations.

### Core space characterization and dynamics

Core space is often operationally defined as the 50% isopleth, but studies rarely include a quantitative assessment of whether individuals actually exhibit concentrated use relative to their overall movements or home ranging patterns [9, 16, 20]. This distinction may be particularly important to consider for juveniles, as it has been shown that in some species, individuals may exhibit flexibility in behaviors and diet throughout their early-life until they adopt a more stable strategy [5]. By applying an individual-based quantitative method, we demonstrated that for all individuals, in bi-monthly temporal windows, core space use could be identified and quantified in the utilization distributions.

Our results identified the spatial locations within the SSL home range that were quantitatively considered ‘core’. Overall, these core spaces tended to be associated with known haulouts, which complements previous studies of movement and haulout usage for this age-class [4]. Since we included land and sea locations to quantify juveniles’ total UD, we cannot determine whether this concentrated use near haulouts reflects animals on land, or animals’ rafting or swimming in the nearshore waters [35]. However, this approach did also identify core spaces in several at-sea locations, suggesting these habitats are as biologically important to juveniles as the areas near haulouts. In Prince William Sound, core spaces were identified in areas such as Culross Pass, a 0.5-2 km narrow pass between Culross Island and the mainland, and in the northwest fjords (e.g. College Fjord, Harriman Fjord, Valdez Arm). Similarly, core spaces in the Kenai Fjords region also extended up into the heads of several fjords (e.g. Northwestern Fjord). This observation differs from previous assessments that suggested juveniles and pups in Prince William Sound rarely ventured into the northern fjords [4]. Tracking studies on ringed seals (*Pusa hispida*) have shown that fjords can act as a spatial refuge during adverse oceanic conditions [81], and fjords may also provide opportunities for seasonal exploitation of ephemeral but predictable resources for many marine predators [82, 83]. While the quantitative characteristics of core may be difficult to compare across studies that differ in data collection [80] or behavioral contexts [20, 53], our results highlight that core space may be important to consider as an ecological indicator, and that visualization of these spaces may identify areas of use that were previously overlooked.

When looking at the characteristics of individuals’ core space, we found that size, proximity to haulouts, and intensity of use varied seasonally by region, with similar sex-specific patterns to those observed for home range characteristics. In the Kenai Fjords-Gulf of Alaska region, core sizes were smaller, were closer to haulouts, and intensity of use relative to the home range size was greater in the summer (May–August) than in the rest of the year. Size, proximity and intensity of core spaces in Prince William Sound, and for individuals whose home ranges spanned both regions, did not significantly exhibit any seasonal variation. The seasonal pattern in the Kenai Fjords-Gulf of Alaska complements previous studies that showed summer home ranges were smaller than winter home ranges for adult females near Kodiak Island [38], which is part of the Kenai Fjords-Gulf of Alaska region in our study. However, it is unclear why these patterns were not observed in Prince William Sound animals. For upper-trophic level predators, prey distributions can be a driver of core space characteristics. For example, home range size for individual male lynx was influenced by conspecific density, but the size of their core space was influenced by prey density [15]. Similarly, adult Steller sea lions exhibit increased offshore foraging in the winter and concentrated use near haulouts in the summer, which may be in response to shifts in prey resources [82, 83]. The close proximity of multiple haulouts (median 3 haulouts within 20 km of a given haulout) in the Kenai Fjords region in this study could facilitate individuals’ ability to respond to seasonal changes in resource distribution and expand or contract their space use accordingly. In contrast, in Prince William Sound haulouts are farther apart (median 1.5 haulouts within 20 km of a given haulout) and movements tend to remain with discrete spatial clusters [4], suggesting variation in response to seasonal pulses of prey might be constrained. While we cannot confirm in the current study the mechanism driving the region-specific seasonal pattern, it highlights an interesting avenue for further exploration.

## Conclusions

This study represents the first characterization of the population, and individual utilization distributions for endangered, juvenile Steller sea lions in the eastern Gulf of Alaska. Utilization distributions derived from movement data have provided key insights for the management of marine megafauna in terms of identifying spatial overlap with bycatch [20] or protected areas [53]. It may also be important to consider the spatial overlap and encounter probabilities between predators and prey by comparing utilization distributions generated from tracking data of each [1, 37]. Previous bio-telemetry data from juvenile Steller sea lions has suggested that predation is a major cause of mortality for this age-class in the Kenai Fjords-Prince William Sound region [31, 32] and there is preliminary support that predation risk may influence juvenile behavior [84]. Quantifying the overlap between juvenile Steller sea lion space use and the space use of their potential predators such as transient killer whales (*Orcinus orca*) [85] and sharks [46], may be important for gaining a better understanding of the role predators play in shaping animal movements, as was shown in a study utilizing simultaneous tracking data from narwhal (*Monodon monoceros*) and killer whales [37]. Understanding the areas of biological importance and seasonal variation in space use for this age-class will therefore allow for future assessments of ecological dynamics, such as predator-prey interactions, and inform the management of a listed population.

## Additional file

Additional file 1: **Table S1.** Summary of tag deployments and individual animal information for individuals included in final UD analysis (*n* = 84). (DOCX 45 kb)

## Abbreviations

GAMMs: Generalized additive mixed-effects models
GLMMs: Generalized linear mixed-effects models
kde: Kernel density estimate
KFGOA: Kenai Fjords/Gulf of Alaska
PWS: Prince William Sound
SDR: Satellite data recorder
SSL: Steller sea lion
UD: Utilization distribution
wDPS: Western Distinct Population Segment
