# Supplementary material for: Juvenile Steller sea lion (Eumetopias jubatus) utilization distributions in the Gulf of Alaska
Source: Mov Ecol. 2018 May 15;6:6. doi: 10.1186/s40462-018-0124-6 (PMC5952600; doi:10.1186/s40462-018-0124-6)
Supplement: Supplementary file 1 — Table S1. Summary of tag deployments and individual animal information for individuals included in final UD analysis (n = 84). (DOCX 45 kb) [file 40462_2018_124_MOESM1_ESM.docx]

**Table S1:** Summary of tag deployments and individual animal information for individuals included in final UD analysis (n=84).

| **Animal ID** | **Sex** | **LHX** | **Release Date** | **Capture Site** | **Release Site** | **Last Loc. Date** | **Num. Days** | **Age* (mo)** | **Mass* (kg)** | **Raw Locations** | **Pseudo-locations** | **Ratio of Pseudo: Raw** |  | |  | |
| --- | --- | --- | --- | --- | --- | --- | --- | --- | --- | --- | --- | --- | --- | --- | --- | --- |
| TJ01 | M | No | 9/10/2003 | Cape Res | Cape Res | 11/30/2003 | 81 | 14 | 121 | 802 | 968 | 1.21 |  |  |  |  |
| TJ02 | F | No | 10/2/2003 | Cape Res | Cape Res | 10/16/2003 | 14 | 15 | 104 | 103 | 160 | 1.55 |  |  |  |  |
| TJ03 | M | No | 12/8/2003 | Proc Rock | Proc Rock | 4/15/2004 | 129 | 17 | 134 | 1121 | 1512 | 1.35 |  |  |  |  |
| TJ04 | F | No | 12/8/2003 | Proc Rock | Proc Rock | 5/5/2004 | 149 | 17 | 145 | 1250 | 1529 | 1.22 |  |  |  |  |
| TJ05 | F | No | 5/26/2004 | GI-PWS | GI-PWS | 6/27/2004 | 32 | 23 | 129 | 262 | 321 | 1.23 |  |  |  |  |
| TJ06 | F | No | 5/26/2004 | GI-PWS | GI-PWS | 7/5/2004 | 40 | 23 | 140 | 425 | 467 | 1.1 |  |  |  |  |
| TJ07 | F | No | 5/26/2004 | GI-PWS | GI-PWS | 6/27/2004 | 32 | 23 | 166 | 296 | 362 | 1.22 |  |  |  |  |
| TJ08 | M | No | 5/26/2004 | GI-PWS | GI-PWS | 6/16/2004 | 21 | 23 | 176 | 183 | 228 | 1.25 |  |  |  |  |
| TJ09 | M | No | 9/28/2004 | Cape Res | Low Pt | 2/20/2005 | 145 | 15 | 134 | 1512 | 1728 | 1.14 |  |  |  |  |
| TJ10 | F | No | 9/28/2004 | Cape Res | Low Pt | 12/21/2004 | 84 | 15 | 107 | 759 | 937 | 1.23 |  |  |  |  |
| TJ11 | M | No | 9/28/2004 | PtEL-PWS | Pt.EL-PWS | 2/11/2005 | 136 | 15 | 114 | 1369 | 1631 | 1.19 |  |  |  |  |
| TJ12 | M | No | 9/28/2004 | GI-PWS | GI-PWS | 3/8/2005 | 161 | 15 | 135 | 1519 | 1923 | 1.27 |  |  |  |  |
| TJ13 | M | No | 4/28/2005 | GI-PWS | Low Pt | 6/16/2005 | 49 | 22 | 186 | 501 | 579 | 1.16 |  |  |  |  |
| TJ14 | F | No | 4/28/2005 | GI-PWS | Low Pt | 6/13/2005 | 46 | 22 | 144 | 460 | 530 | 1.15 |  |  |  |  |
| TJ15 | M | No | 4/28/2005 | GI-PWS | Low Pt | 7/26/2005 | 89 | 22 | 145 | 838 | 1051 | 1.25 |  |  |  |  |
| TJ16 | M | No | 4/28/2005 | GI-PWS | Low Pt | 6/19/2005 | 52 | 22 | 190 | 581 | 602 | 1.04 |  |  |  |  |
| TJ17 | M | No | 8/4/2005 | GI-PWS | Low Pt | 10/9/2005 | 66 | 13 | 128 | 632 | 660 | 1.04 |  |  |  |  |
| TJ18 | M | No | 8/4/2005 | GI-PWS | Low Pt | 9/9/2005 | 36 | 13 | 111 | 406 | 428 | 1.05 |  |  |  |  |
| TJ19 | F | No | 8/4/2005 | GI-PWS | Low Pt | 9/1/2005 | 28 | 13 | 116 | 297 | 329 | 1.11 |  |  |  |  |
| TJ20 | F | No | 8/4/2005 | GI-PWS | Low Pt | 10/28/2005 | 85 | 13 | 105 | 993 | 886 | 0.89 |  |  |  |  |
| TJ21 | F | No | 11/22/2005 | N-PWS | Low Pt | 3/27/2006 | 125 | 17 | 138 | 1338 | 1493 | 1.12 |  |  |  |  |
| TJ22 | F | Yes | 11/22/2005 | N-PWS | Low Pt | 3/27/2006 | 125 | 17 | 131 | 2159 | 1492 | 0.69 |  |  |  |  |
| TJ23 | M | Yes | 11/22/2005 | N-PWS | Low Pt | 2/2/2006 | 72 | 17 | 136 | 1141 | 855 | 0.75 |  |  |  |  |
| TJ24 | M | Yes | 4/17/2006 | GI-PWS | Low Pt | 7/5/2006 | 79 | 22 | 180 | 1292 | 924 | 0.72 |  |  |  |  |
| TJ25 | M | Yes | 4/17/2006 | GI-PWS | Low Pt | 6/29/2006 | 73 | 22 | 139.8 | 808 | 851 | 1.05 |  |  |  |  |
| TJ26 | M | Yes | 4/17/2006 | GI-PWS | Low Pt | 7/31/2006 | 105 | 22 | 171.6 | 1290 | 1239 | 0.96 |  |  |  |  |
| TJ27 | M | Yes | 4/17/2006 | GI-PWS | Low Pt | 6/26/2006 | 70 | 22 | 149.4 | 1185 | 819 | 0.69 |  |  |  |  |
| TJ32 | M | Yes | 10/10/2007 | GI-PWS | Low Pt | 2/23/2008 | 136 | 15 | 147 | 1454 | 1389 | 0.96 |  |  |  |  |
| TJ33 | M | Yes | 10/10/2007 | GI-PWS | Low Pt | 2/11/2008 | 124 | 15 | 134 | 1215 | 1456 | 1.2 |  |  |  |  |
| TJ34 | M | Yes | 10/9/2007 | GI-PWS | Low Pt | 3/31/2008 | 174 | 15 | 110 | 1315 | 2056 | 1.56 |  |  |  |  |
| TJ35 | M | Yes | 10/9/2007 | GI-PWS | Low Pt | 2/8/2008 | 122 | 15 | 146 | 1951 | 1342 | 0.69 |  |  |  |  |
| TJ36 | M | Yes | 10/9/2007 | GI-PWS | Low Pt | 2/23/2008 | 137 | 15 | 113 | 1715 | 1609 | 0.94 |  |  |  |  |
| TJ37 | M |  | 10/10/2007 | GI-PWS | Low Pt | 1/13/2008 | 95 | 15 | 131 | 397 | 321 | 0.81 |  |  |  |  |
| TJ38 | M | Yes | 4/29/2008 | GI-PWS | Low Pt | 5/28/2008 | 29 | 22 | 197.6 | 215 | 346 | 1.61 |  |  |  |  |
| TJ39 | M | Yes | 4/29/2008 | GI-PWS | Low Pt | 6/12/2008 | 44 | 22 | 164.8 | 167 | 338 | 2.02 |  |  |  |  |
| TJ40 | F | Yes | 4/29/2008 | GI-PWS | Low Pt | 6/20/2008 | 52 | 22 | 99.6 | 336 | 614 | 1.83 |  |  |  |  |
| TJ41 | M | Yes | 4/29/2008 | GI-PWS | Low Pt | 6/26/2008 | 58 | 22 | 162.6 | 361 | 681 | 1.89 |  |  |  |  |
| TJ43 | F | Yes | 11/12/2008 | GI-PWS | Low Pt | 4/3/2009 | 142 | 16 | 145.2 | 993 | 1704 | 1.72 |  |  |  |  |
| TJ44 | M | Yes | 11/11/2008 | GI-PWS | Low Pt | 11/21/2008 | 10 | 16 | 130.5 | 100 | 125 | 1.25 |  |  |  |  |
| TJ45 | M | Yes | 11/12/2008 | GI-PWS | Low Pt | 1/5/2009 | 54 | 16 | 157.4 | 580 | 651 | 1.12 |  |  |  |  |
| TJ46 | M | Yes | 11/11/2008 | GI-PWS | Low Pt | 2/17/2009 | 98 | 16 | 134 | 888 | 990 | 1.11 |  |  |  |  |
| TJ47 | F | Yes | 11/11/2008 | GI-PWS | Low Pt | 1/17/2009 | 67 | 16 | 102.6 | 724 | 808 | 1.12 |  |  |  |  |
| TJ48 | M | Yes | 11/11/2008 | GI-PWS | Low Pt | 6/14/2009 | 215 | 16 | 126.2 | 1160 | 2316 | 2 |  |  |  |  |
| TJ51 | F | Yes | 7/21/2009 | GI-PWS | Low Pt | 8/15/2009 | 25 | 13 | 100.4 | 157 | 244 | 1.55 |  |  |  |  |
| TJ52 | M | Yes | 7/29/2009 | GI-PWS | SMC | 10/12/2009 | 75 | 25 | 160.6 | 656 | 824 | 1.26 |  |  |  |  |
| TJ54 | M | Yes | 7/21/2009 | GI-PWS | Low Pt | 8/11/2009 | 21 | 13 | 73.2 | 87 | 242 | 2.78 |  |  |  |  |
| TJ55 | M | Yes | 7/29/2009 | GI-PWS | SMC | 11/10/2009 | 104 | 13 | 80.2 | 944 | 1238 | 1.31 |  |  |  |  |
| TJ56 | M | Yes | 11/24/2010 | GI-PWS | Low Pt | 12/20/2010 | 26 | 17 | 109.8 | 456 | 303 | 0.66 |  |  |  |  |
| TJ57 | M | Yes | 11/24/2010 | PI-PWS | Low Pt | 4/9/2011 | 136 | 17 | 111.4 | 2389 | 1623 | 0.68 |  |  |  |  |
| TJ58 | M | Yes | 11/23/2010 | PI-PWS | Low Pt | 2/12/2011 | 81 | 17 | 110 | 1600 | 969 | 0.61 |  |  |  |  |
| TJ59 | M | Yes | 11/23/2010 | PI-PWS | Low Pt | 4/18/2011 | 146 | 17 | 114 | 2048 | 1752 | 0.86 |  |  |  |  |
| TJ60 | F | Yes | 12/20/2010 | PI-PWS | Low Pt | 1/2/2011 | 13 | 18 | 114 | 153 | 155 | 1.01 |  |  |  |  |
| TJ62 | F | Yes | 6/29/2011 | GI-PWS | Low Pt | 8/2/2011 | 34 | 12 | 94 | 642 | 405 | 0.63 |  |  |  |  |
| TJ63 | M | Yes | 6/22/2011 | GI-PWS | Low Pt | 7/16/2011 | 24 | 12 | 108 | 358 | 239 | 0.67 |  |  |  |  |
| TJ64 | F | Yes | 6/22/2011 | GI-PWS | Low Pt | 7/18/2011 | 26 | 12 | 113 | 369 | 305 | 0.83 |  |  |  |  |
| TJ65 | M | No | 6/15/2011 | GI-PWS | Low Pt | 7/30/2011 | 45 | 12 | 108.2 | 383 | 479 | 1.25 |  |  |  |  |
| LHX2-03 | F | Yes | 5/14/2014 | GI-PWS | Low Pt | 7/11/2014 | 58 | 22 | 104 | 570 | 689 | 1.21 |  |  |  |  |
| LHX2-04 | F | Yes | 5/14/2014 | GI-PWS | Low Pt | 7/3/2014 | 50 | 22 | 117 | 510 | 582 | 1.14 |  |  |  |  |
| LHX2-05 | F | Yes | 5/14/2014 | GI-PWS | Low Pt | 6/26/2014 | 43 | 22 | 87 | 508 | 507 | 1 |  |  |  |  |
| LHX2-06 | F | Yes | 5/14/2014 | GI-PWS | Low Pt | 7/11/2014 | 58 | 22 | 74 | 745 | 685 | 0.92 |  |  |  |  |
| LHX2-07 | F | Yes | 5/14/2014 | GI-PWS | Low Pt | 7/11/2014 | 58 | 22 | 75 | 705 | 689 | 0.98 |  |  |  |  |
| LHX2-08 | F | Yes | 5/14/2014 | GI-PWS | Low Pt | 7/11/2014 | 58 | 12 | 77 | 845 | 689 | 0.82 |  |  |  |  |
| LHX2-09 | F | Yes | 8/28/2014 | GI-PWS | Low Pt | 9/27/2014 | 30 | 14 | 74 | 110 | 247 | 2.25 |  |  |  |  |
| LHX2-10 | F | Yes | 8/28/2014 | GI-PWS | Low Pt | 12/25/2014 | 119 | 26 | 103 | 1284 | 1326 | 1.03 |  |  |  |  |
| LHX2-11 | F | Yes | 8/28/2014 | GI-PWS | Low Pt | 12/11/2014 | 105 | 26 | 105 | 639 | 438 | 0.69 |  |  |  |  |
| FR010 | M | No | 7/28/2004 | GI-PWS | GI-PWS | 9/28/2004 | 62 | 25 | 119 | 734 | 730 | 0.99 |  |  |  |  |
| FR011 | F | No | 7/28/2004 | GI-PWS | GI-PWS | 8/9/2004 | 12 | 25 | 99 | 83 | 132 | 1.59 |  |  |  |  |
| FR013 | F | No | 7/29/2004 | GI-PWS | GI-PWS | 8/13/2004 | 15 | 25 | 101 | 126 | 175 | 1.39 |  |  |  |  |
| FR014 | F | No | 7/29/2004 | GI-PWS | GI-PWS | 8/27/2004 | 29 | 25 | 79 | 222 | 271 | 1.22 |  |  |  |  |
| FR63 | F | No | 6/1/2011 | GI-PWS | GI-PWS | 7/20/2011 | 49 | 12 | 76 | 524 | 589 | 1.12 |  |  |  |  |
| FR65 | M | No | 6/1/2011 | GI-PWS | GI-PWS | 7/20/2011 | 49 | 12 | 80.4 | 602 | 577 | 0.96 |  |  |  |  |
| FR66 | M | No | 8/8/2012 | GI-PWS | GI-PWS | 9/13/2012 | 36 | 14 | #N/A | 288 | 138 | 0.48 |  |  |  |  |
| FR67 | F | No | 8/8/2012 | GI-PWS | GI-PWS | 9/5/2012 | 28 | 14 | #N/A | 634 | 318 | 0.5 |  |  |  |  |
| FR68 | M | No | 8/8/2012 | GI-PWS | GI-PWS | 11/2/2012 | 86 | 14 | #N/A | 1644 | 1018 | 0.62 |  |  |  |  |
| FR69 | M | No | 8/8/2012 | GI-PWS | GI-PWS | 8/31/2012 | 23 | 26 | #N/A | 499 | 263 | 0.53 |  |  |  |  |
| FR70 | M | No | 8/9/2012 | GI-PWS | GI-PWS | 8/22/2012 | 13 | 14 | #N/A | 237 | 151 | 0.64 |  |  |  |  |
| SSL267PWS01 | M | No | 11/7/2001 | GI-PWS | GI-PWS | 5/31/2002 | 205 | 17 | 133.9 | 1841 | 2453 | 1.33 |  |  |  |  |
| SSL270PWS01 | F | No | 11/8/2001 | GI-PWS | GI-PWS | 3/24/2002 | 136 | 17 | 96.4 | 864 | 1633 | 1.89 |  |  |  |  |
| SSL274PWS01 | M | No | 11/9/2001 | PI-PWS | PI-PWS | 4/29/2002 | 171 | 17 | 178.2 | 2142 | 2056 | 0.96 |  |  |  |  |
| SSL275PWS01 | F | No | 11/9/2001 | PI-PWS | PI-PWS | 6/10/2002 | 213 | 17 | 123.2 | 2128 | 2553 | 1.2 |  |  |  |  |
| SSL276PWS01 | M | No | 11/9/2001 | PI-PWS | PI-PWS | 5/29/2002 | 201 | 17 | 148.8 | 2144 | 2409 | 1.12 |  |  |  |  |
| SSL77PWS00^*^ | F | No | 4/23/2000 | GI-PWS | GI-PWS | 5/19/2000 | 26 | 12.5 | 98 | 115 | 295 | 2.57 |  |  |  |  |
| SSL81PWS00^*^ | F | No | 4/25/2000 | GI-PWS | GI-PWS | 6/11/2000 | 47 | 12.5 | 83 | 204 | 547 | 2.68 |  |  |  |  |
| SSL85PWS00 | F | No | 4/26/2000 | N-PWS | N-PWS | 6/19/2000 | 54 | 24.5 | 134.5 | 213 | 628 | 2.95 |  |  |  |  |
| **AVERAGE** | -- |  | -- |  | -- | -- | **77** | **--** | **123.3** | **815.4** | **866.6** | **1.19** |  |  |  |  |
| Animals excluded from final analysis because they didn't meet criteria of a minimum of 50 locations per bi-month criteria for kde calculation, ratio of pseudolocations to raw locations > 3, or isopleths wouldn't calculate due to area being < 1km^2.^ (n=4) | | | | | | | | | | | | | |  | |  |
| FR015 | M |  | 7/29/2004 | GI-PWS | GI-PWS | 8/13/2004 | 15 | 13 | 99 | 80 | 157 | 1.96 |  |  |  |  |
| FR64 | F |  | 6/1/2011 | GI-PWS | GI-PWS | 7/10/2011 | 39 | 12 | 72.5 | 360 | 444 | 1.23 |  |  |  |  |
| TJ50 | M |  | 7/29/2009 | GI-PWS | SMC | 9/1/2009 | 34 | 13 | 136 | 249 | 361 | 1.45 |  |  |  |  |
| LHX2-12 | F |  | 8/28/2014 | GI-PWS | Low Pt | 12/26/2014 | 120 | 26 | 78 | 323 | 1258 | 3.89 |  |  |  |  |

******* *age and mass at release. For animals SSL77PWS00 and SSL81PWS00 release age was 10.5 months but each had 1 bi-month period >12 mo old.*

***Prince William Sound*** *GI-PWS= Glacier Island; PI-PWS = Perry Island, PtEL-PWS = Pt. Elrington, N = The Needle; Proc Rock = Procession Rocks*

***Kenai Fjords*** *Low Pt = Lowell Point; SMC = Seward Marine Center/ASLC; Cape Res = Cape Resurrection*
